# An intelligent medical guidance and recommendation model driven by patient-physician communication data

**DOI:** 10.3389/fpubh.2023.1098206

**Published:** 2023-01-26

**Authors:** Jusheng Liu, Chaoran Li, Ye Huang, Jingti Han

**Affiliations:** ^1^School of Economics and Management, Shanghai University of Political Science and Law, Shanghai, China; ^2^School of Economics and Management, Shanghai University of Sport, Shanghai, China; ^3^School of Information Management and Engineering, Shanghai University of Finance and Economics, Shanghai, China; ^4^Shanghai Financial Intelligent Engineering Technology Research Center, Shanghai, China

**Keywords:** healthcare, recommendation system, natural language processing, text analytics, patient-physician communication data

## Abstract

Based on the online patient-physician communication data, this study used natural language processing and machine learning algorithm to construct a medical intelligent guidance and recommendation model. First, based on 16,935 patient main complaint data of nine diseases, this study used the word2vec, long-term and short-term memory neural networks, and other machine learning algorithms to construct intelligent department guidance and recommendation model. Besides, taking ophthalmology as an example, it also used the word2vec, TF-IDF, and cosine similarity algorithm to construct an intelligent physician recommendation model. Furthermore, to recommend physicians with better service quality, this study introduced the information amount of physicians' feedback to the recommendation evaluation indicator as the text and voice service quality. The results show that the department guidance model constructed by long-term and short-term memory neural networks has the best effect. The precision is 82.84%, and the F1-score is 82.61% in the test set. The prediction effect of the LSTM model is better than TextCNN, random forest, K-nearest neighbor, and support vector machine algorithms. In the intelligent physician recommendation model, under certain parameter settings, the recommendation effect of the hybrid recommendation model based on similar patients and similar physicians has certain advantages over the model of similar patients and similar physicians.

## 1. Introduction

At present, with the development of digital medical health, more and more new technologies are being applied to the medical scene (1–3), for example, online medical, adjuvant therapy, and intelligent minimally invasive surgery (4). Although digital medical health and wise medical care have made some progress, the uneven distribution of medical resources is still a serious challenge in China (5). According to the 2020 statistical bulletin on the development of health services in China, the total number of medical and health institutions in China reached 7.74 billion, and the number of medical visits is still at a high level. From the supply side, by the end of 2020, there are 35,394 hospitals in China. Therefore, the mismatch of medical resources is still serious.

To alleviate the unbalanced distribution of medical resources, the governments implement relevant policies around the 'Internet + medical', these policies impel the platform economy and many online health communities (OHCs) to risen, such as Good Doctor, Chunyu Doctor, 39 Health Network, and Dingxiangyuan. Under these platforms, patients can communicate with the physicians, and the physicians can conduct diagnoses and treatment on the patients (6–9). Although online health communities provide more information and convenience to patients, patients still face great difficulties in selecting the department and the physicians. In fact, due to the asymmetric medical knowledge information between physicians and patients, more patients know their disease symptoms, but do not know how to select a department and a physician, especially older people (10). Thus, how to design intelligent medical guidance and recommendation model is important to most patients.

In OHCs, the communication data between physicians and patients has important value (11). In fact, when the patients consult a physician, the patients will disclose their symptoms to the physician. The data which is disclosed by the patients can reflect the patient's symptoms. According to multiple data from patients, the machine learning algorithm is used to learn the text's meaning and predict the department according to the patient's symptoms. Besides, when the patients find an appropriate department, they cannot find the right physician, because there are many physicians in a department, especially in OHCs. At this moment, it is important to recommend an appropriate physician to the patient. The common practice can be divided into three types: similar patients, similar physicians, and similar patients and physicians. Similar patients mean that if the symptoms between two patients are similar, we can recommend one's physician to the other patient. Similar physicians mean that if one patient consults a physician, we can recommend a similar physician to the next patient. Similar patients and physicians mean that we can combine similar patients and similar physicians and determine the final recommended physician.

At present, concerning the department and physician recommendations, previous studies have explored them from different perspectives (12). In terms of department recommendation, Mullenbach et al. (13) combined the attention mechanism and used LSTM to predict patients' disease types. Mao et al. (14) used the undirected multivariate graph neural network (GNN) to construct a relationship model among patients, departments, and prescriptions to recommend the prescription to the patients. Li and Yu (15) employed the multi-filter residual convolutional neural network to explore the problem of the department recommendation. Besides, with the emergence of a new language model, Wang et al. (16) used the BERT model to explore disease diagnosis and department recommendations.

In terms of physician recommendation, Ju and Zhang (17) combined the patients' geographic location and main complaint text and used the word2vec model and cosine similarity to do a personalized recommendation for patients. Mondal et al. (18) used a multilayer graph and considered the patient's trust in doctors to construct a multilayer network recommendation model, which can use a network to store massive heterogeneous information to achieve the goal of personalized recommendations of physicians to patients. Guo et al. (19) recommended doctors with high academic reputations to patients based on the papers published by doctors, media reports, and fund projects obtained by doctors. Based on the patient preferences and doctor online comments, Yang et al. (20) used the intuitionistic fuzzy sets (IFSs) and Bonferroni means (BM) to solve the interdependence of recommended doctors, and this method can improve the diversity and coverage of recommended doctors. Zhang et al. (21) used a latent dirichlet allocation (LDA) model to extract features of patients' preferences and physicians' characteristics to construct a physician recommendation model. Meng and Xiong (22) used the word2vec and LDA model to indicate the physicians' characteristics, and used the cosine similarity of physicians' characteristics to construct a network and obtain the important doctors based on the vector centrality of a network. Besides, with the development of the algorithm, some researchers use the deep learning algorithm to recommend the physician to patients, such as restricted boltzmann machine (RBM)-convolutional neural network (CNN) method (23) and probabilistic matrix factorization integrated with CNN (PMF-CNN) method (24). Furthermore, Xu et al. (25) also considered the doctors' reputation in the doctor recommendation system.

Throughout the above research, it can be seen that existing research has explored the department and physician recommendation separately. However, there are still some gaps in current research. First, current studies used Chinese context data for research rarely, such as Good Doctor. Second, when people use the method of text classification, they do not pay attention to the semantic relevance of the long text in patients' complaint text. Third, current studies recommend departments or physicians separately, they have not constructed intelligent medical guidance and recommendation model that can recommend departments and physicians. Finally, in terms of the evaluating indicator of recommending physicians, most researchers use precision as a single indicator and do not consider the service quality of recommended physicians.

Based on this, our research constructed intelligent medical guidance and recommendation model based on patient-physician communication data. Distinct from the previous research, this research has several innovations and contributions. First of all, our research constructs an intelligent medical guidance and recommendation model driven by patient-physician communication data. Then, our research uses the LSTM algorithm to deal with the semantic relevance of the long text in patients' complaint text. Finally, our research considers service quality as the evaluating indicator for evaluating the recommended physician. This research will help to resolve the mismatch of patient-physician resources, provide some support for old people who do not select the appropriate departments and doctors, and build a harmonious patient-physician relationship in the future.

The remainder of the article is as follows: Section 2 is the intelligent medical department recommendation model; the intelligent medical physician recommendation model is shown in Section 3; we discuss the main research content in Section 4; besides, the research limitations and future research directions are discussed in Section 5; finally, we summarize the conclusion in Section 6.

## 2. Intelligent medical department recommendation model

### 2.1. The process flow of intelligent department guidance

**Figure 1:**
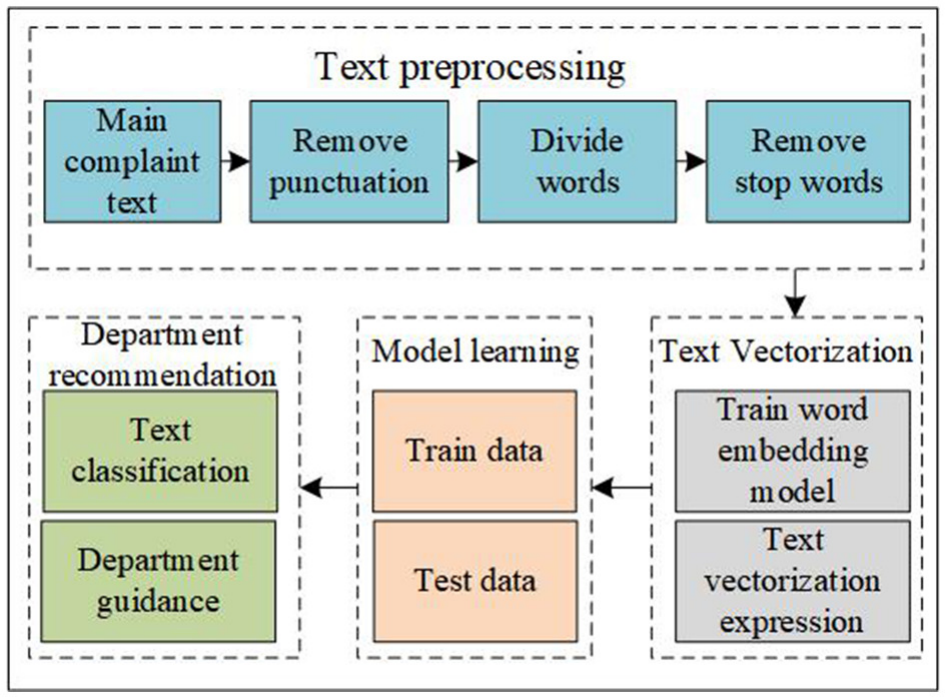
The process flow of intelligent department guidance.

When the patients consult a physician, the patient's complaint text is a kind of description of their symptoms. Classifying the patient's main complaint text can achieve the goal of department recommendation. Therefore, to some degree, intelligent department guidance can be transformed into a text multi-classification problem (26–28). In our research, the data comes from Good Doctor, which is a big online health community in China. We select 16,935 data in Good Doctor randomly and these data belonged to 9 diseases and 8 departments. The diseases contain cataracts, myopia, rhinitis, renal failure, sexual dysfunction, diabetes, abortion, fracture, and infertility. Inside, cataracts and myopia belong to one department named ophthalmology. The process flow of intelligent department guidance is shown in Figure 1.

As shown in Figure 1, first, we need to do a pretreatment on the patient's main complaint text. We need to remove punctuation in the main complaint text, divide the sentence into several words, and remove the stop words. At this time, the sentence is divided into several words without stop words. To measure the meaning of the text, we need to make the text vectorized. In this step, due to the professionalism of medical terms, we need to train a word embedding model and use the vector to indicate the text. After that, we need to use some machine learning and deep learning algorithms to train and test the data. Finally, the department guidance can be transformed into a question of text classification. In our data, different patients have different speaking habits, therefore, when patients consult a physician, some patients speak fewer words such as two words and some patients speak many words such as 1,095 words in our research. The average value of the patients' text length is 88.64 and the median is 50. The patient's main complaint text is shown in Table 1.

**Table 1:** Patient's main complaint text.

**Number**	**Patient main complaint text**	**Disease**	**Department**
1	When I perm on Saturday, the potion enters my eyes. On Monday, I felt that part of my eyes was red and a little swollen, and occasionally a little sore.	Cataract	Ophthalmology
2	At the 17th week of pregnancy, the child's mother was hospitalized for hyperemesis gravida. At present, there is no abnormal phenomenon when the child looks at things, and occasionally the child seems to be less focused.	Myopia	Ophthalmology
3	I have seen many doctors and taken several kinds of medicine, but it has been bad. Both sides are blocked. If I don't use spray, I won't be able to breathe at all.	Rhinitis	Otorhinolaryngology
4	I have a loss of appetite suddenly, vomiting, dark urine, fear of cold, and abdominal distension.	Renal failure	Nephrology
5	Having taken traditional Chinese medicine and Western medicine before, the situation of high sperm deformity rate and low sperm motility has not been significantly improved.	Sexual dysfunction	Urology Surgery
6	Sugar tolerance hasn't been able to control sugar for a week. Is it over the threshold?	Diabetes	Endocrinology
7	Three months after the abortion, the mixed echo blood flow signal of the uterine cavity was rechecked.	Induced abortion	Gynecology and obstetrics
8	At present, after conservative treatment for 2 months, I can move. That is, the meniscus injury is not so swollen, and the lower pedal can't reach the full angle. For the first time, the anterior cruciate ligament was partially torn. Is this injury surgery or conservative treatment?	Fracture	Orthopedics
9	Hello, Doctor Li, I communicated with you about threatened abortion before, and now it's definite ectopic pregnancy! There is a problem. Now the doctor says that salpingectomy will affect the later in vitro fertilization. thank you.	Infertility	Reproduction

**Figure 2:**
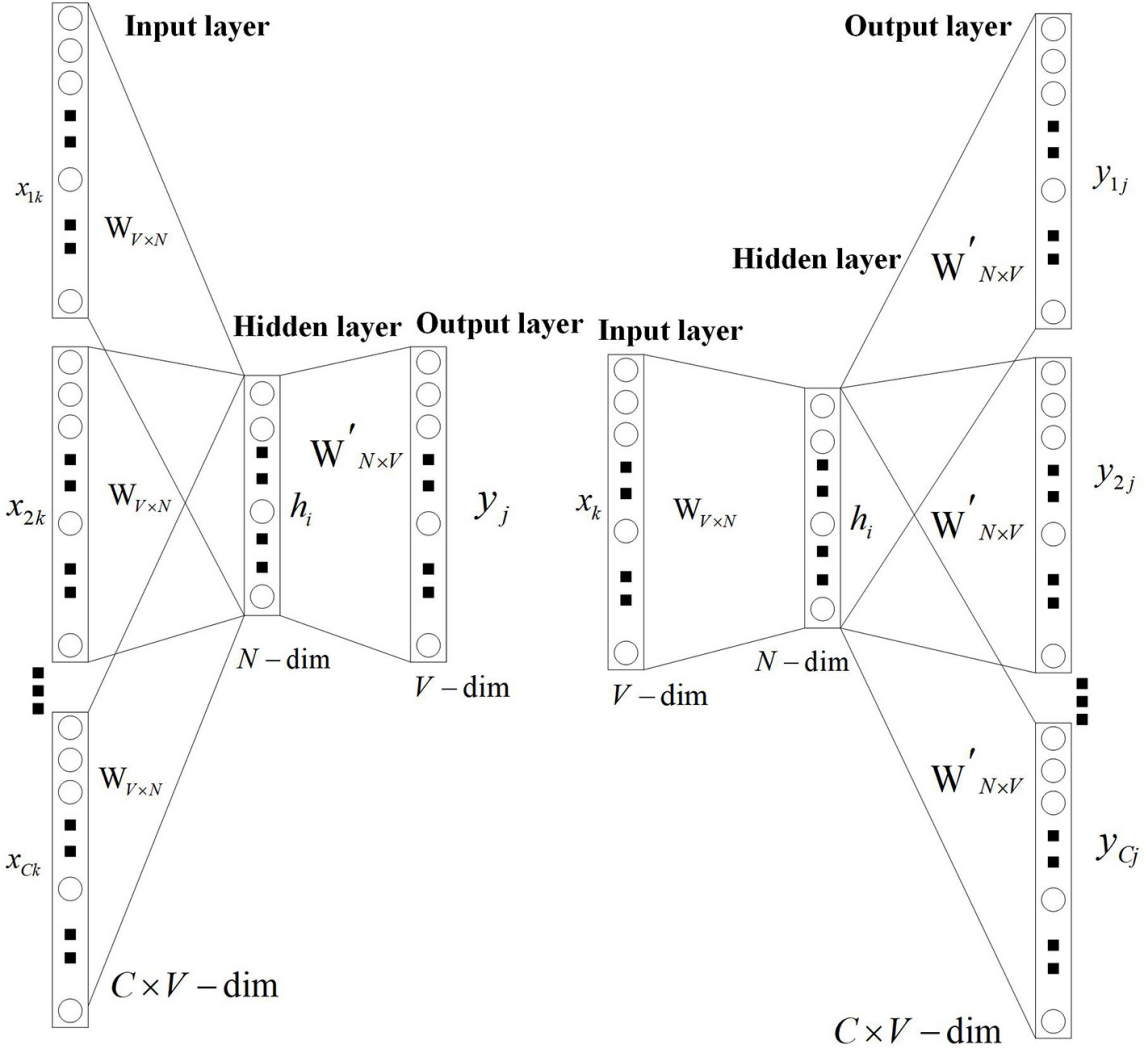
Word vector model.

In the word embedding model, we need to do a text vectorization. In this part, the usual model is the word2vec model. The word2vec model has two forms: skip-grams (29) and continuous bag of words (CBOW) (30). The skip-grams model can infer the semantics of the context by using the semantics of the headword, while the CBOW model can infer the semantics of the headword by using the semantics of the context. The word vector model is shown in Figure 2 (31).

**Figure 3:**
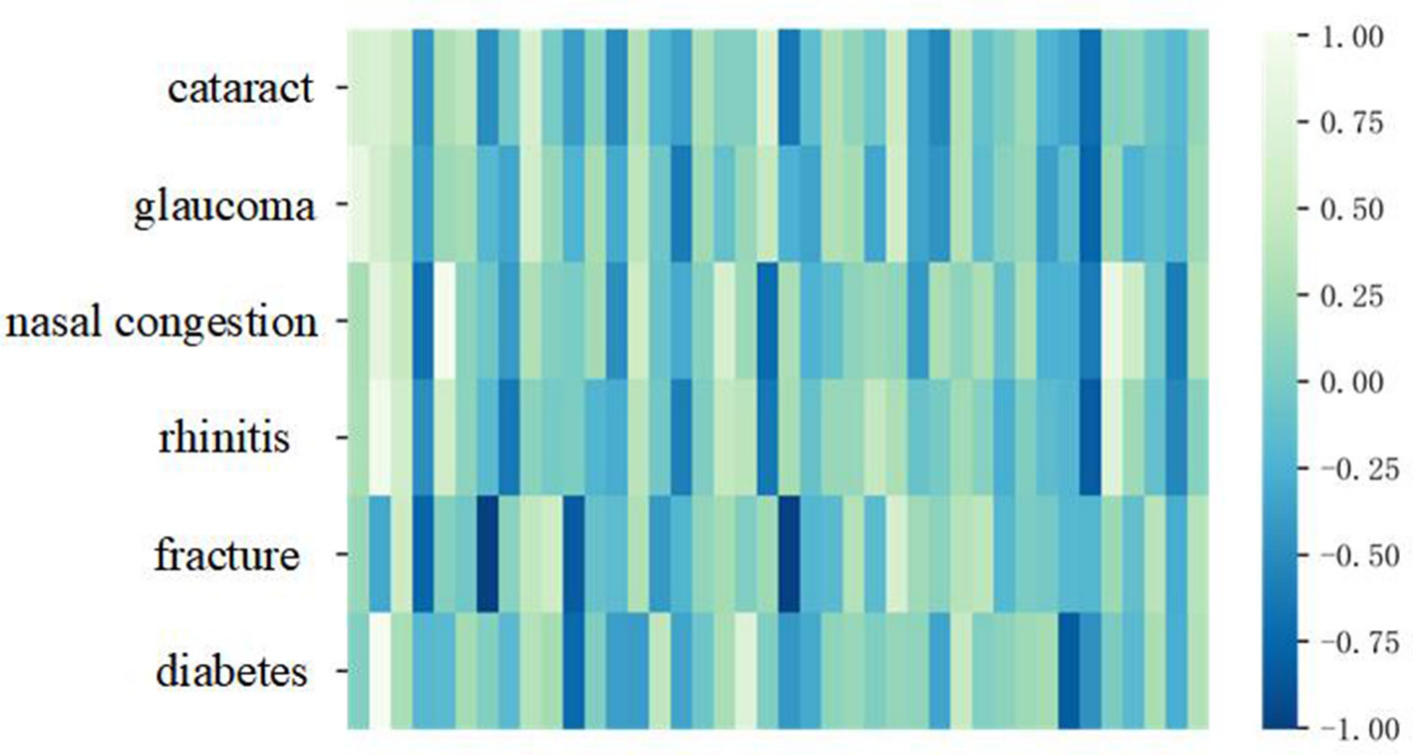
The result of pre-training word vector.

In our research, to make the word vector model have rich and accurate semantic expression ability, we introduce the patient-physician communication on Good Doctor, medical books, and other corpora to the model. To show the model effect intuitively, we train a 40-dimension Skip-Grams model and selected six diseases to do a test. The six diseases are cataracts, glaucoma, nasal congestion, rhinitis, fracture, and diabetes. The result of the pre-training word vector is shown in Figure 3.

It can be seen from Figure 3, the colors of cataracts and glaucoma, nasal congestion, and rhinitis expressed in vectorization are similar under the same dimension in word vector space, while fractures and diabetes differ greatly in vectorization space expression. This result shows that the pre-trained word vector model is more accurate and effective.

**Figure 4:**
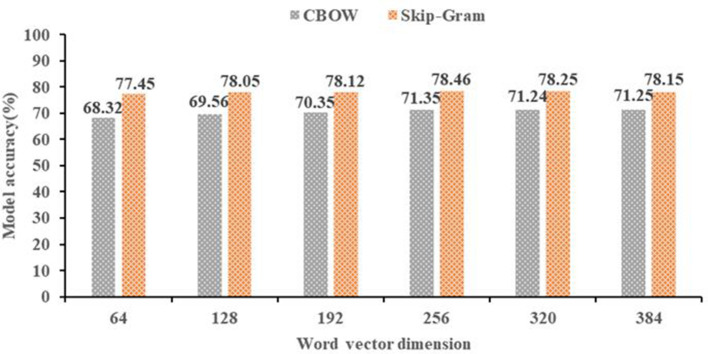
Word vector dimension.

In terms of the dimension of the word vector, we need to determine the appropriate dimension for our model. If the dimension of the word vector is too big, the cost of computers will increase, while the dimension of the word vector is small, the word vector cannot express the meaning of the word well. Thus, it is important to determine the dimension of the word vector. To quickly determine the dimension, we use a classification algorithm named K-Nearest Neighbor (KNN) to determine the final dimension of the word vector. The final result is shown in Figure 4.

As shown in Figure 4, the horizontal axis is the word vector dimension, the vertical axis is the model accuracy of text classification. We can see that the accuracy of the 256 dimensions in the CBOW model is 71.35%, and the accuracy of the 256 dimensions in the skip-grams model is 78.46%. Therefore, the 256 dimension of the skip-grams model is the best word vector.

**Figure 5:**
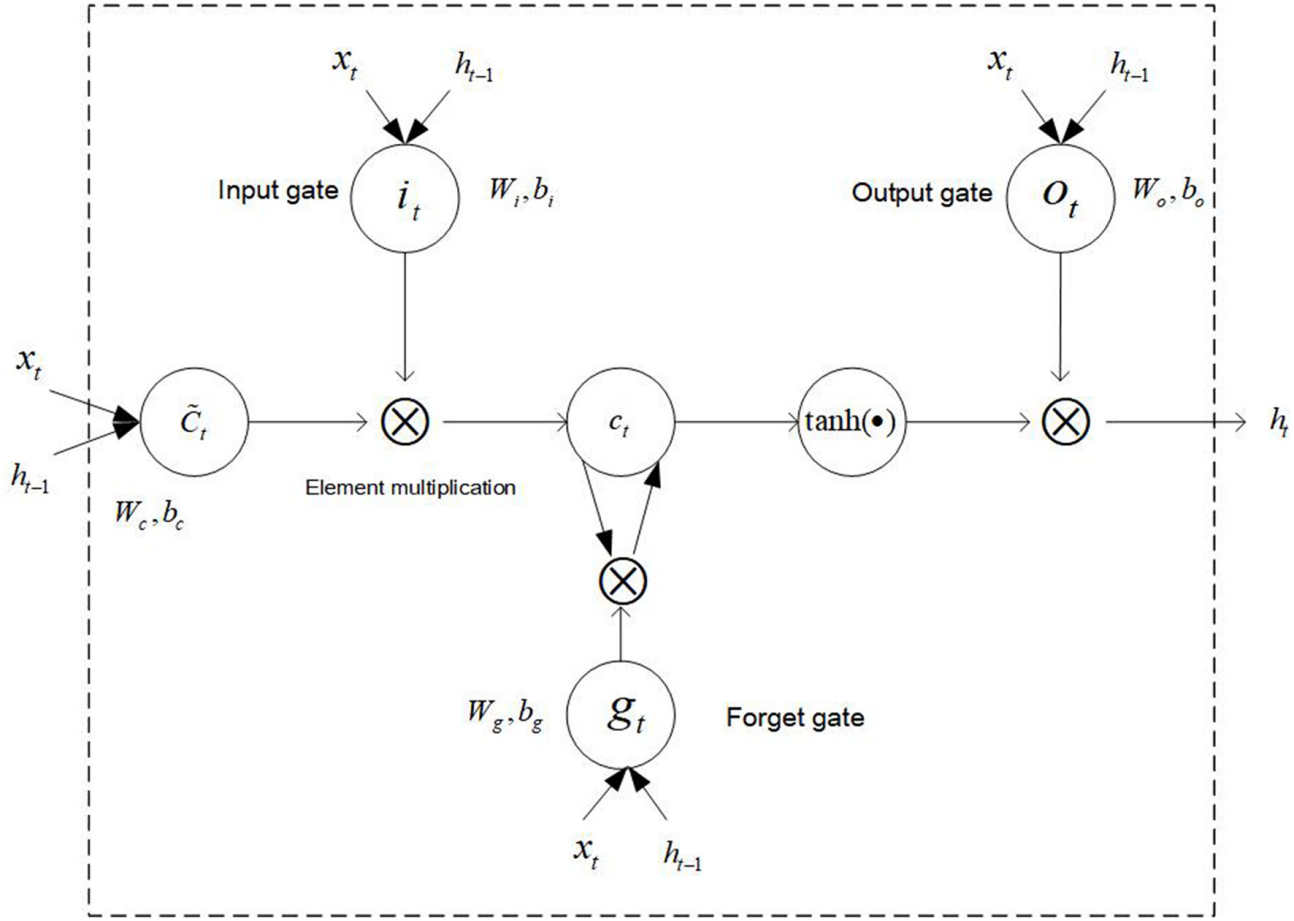
The schematic diagram of the LSTM model.

After training the word2vec model and determining the dimension of the word vector, it is needed to use deep learning or machine learning algorithm to train and test the data. Because the patients' main complaint text of their symptoms is long, the long short-term memory (LSTM) algorithm can deal with the context semantic relationship well based on its advantage of memory function. Therefore, we select the LSTM algorithm to do the department recommendation. The schematic diagram of the LSTM model is shown in Figure 5 (32).

In LSTM, the information processing flow is as follows: (1) Discard information. The information is discarded and retained by the sigmoid function through the forget gate of LSTM.


(1) $$\begin{array}{l} g_{t}=\sigma \left(W_{g}\left[h_{t-1},x_{t}\right]+b_{g}\right) \end{array}$$

Where *g*_*t*_ means the proportion of discarded information, σ is the sigmoid function, *W*_*g*_ indicates the weight of the forget gate, and *b*_*g*_ is the bias term of the forget gate.

(2) Update information. The retained information is updated through the input gate, and then a new candidate vector is created by a layer and added to the state.


(2) $$\begin{array}{l} i_{t}=\sigma \left(W_{i}\left[h_{t-1},x_{t}\right]+b_{i}\right) \end{array}$$

(3) $$\begin{array}{l} \tilde{C_{t}}=tanh\left(W_{c}\left[h_{t-1},x_{t}\right]+b_{c}\right) \end{array}$$

Where *i*_*t*_ is the weight of the updated door, *b*_*t*_ is the bias term of the update gate, *tanh* is the hyperbolic tangent function, *W*_*c*_ indicates the updating candidate values, *b*_*c*_ indicates the updating the bias term of the candidate values, and $\tilde{C_{t}}$ is the candidate value.

(3) Update the cell status. Multiplying the old state and the discard function *g*_*t*_, further update each status, and the updated status value is *C*_*t*_.


(4) $$\begin{array}{l} C_{t}=g_{t}C_{t-1}+i_{t}\tilde{C_{t}} \end{array}$$

(4) Determine output status. The sigmoid function can determine which part of the cell state will be output. The cell state will be processed by *tanh* function, and the processed result will be multiplied by the sigmoid function in gate output to obtain the final output result.


(5) $$\begin{array}{l} O_{t}=\sigma \left(W_{o}\right)\left[h_{t-1},x_{t}\right]+b_{o} \end{array}$$

(6) $$\begin{array}{l} h_{t}=O_{t}tanhC_{t} \end{array}$$

Where *W*_*o*_ is a function that can update the weight, *b*_*o*_ can update the bias term of the output value, and *h*_*t*_ is the final output value.

**Figure 6:**
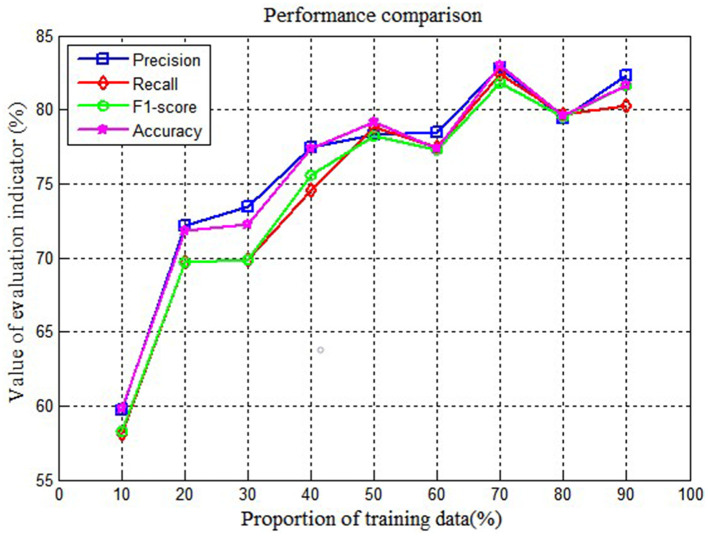
Performance comparison on different proportions of training data.

During the process of the parameter selection process, different proportions of training data may influence the performance of the model. To select the suitable parameters, this study compares the performance of the LSTM model under the different proportions of training data, the specific result is shown in Figure 6.

As shown in Figure 6, it can be found that when the proportion of training data is 70% and the proportion of testing data is 30%, the performance of LSTM is best. Thus, we fix the proportion of training data at 70% and testing data at 30%.

To measure the performance of LSTM in department recommendation, we select machine learning and the deep learning algorithm to do a test. The comparison algorithm contains text CNN (TextCNN), Random Forest, KNN, and SVM algorithms. All algorithms have been dealt with 10-fold cross-validation, and the average result of five times is taken as the final result. Since the LSTM and TextCNN algorithms need to input a matrix, therefore, to keep reasonable input sentence length, the sentence length of LSTM and TextCNN can be set to the median of 75% of all sentence lengths, and the final sentence length is around 110. The input of random forest, KNN, and SVM algorithms is not a matrix, thus the sentence length is not processed. The related parameter settings are shown in Table 2.

**Table 2:** The related parameter settings.

		**Parameter value**				
**Parameter name**	**Parameter meaning**	**LSTM**	**TextCNN**	**Random Forest**	**KNN**	**SVM**
Max_len	Maximum sentence length	110	110	unlimited	unlimited	unlimited
Embedding_dim	Dimension of word vector	256	256	256	256	256
LSTM_layer_num	Circulating layer neuron	256	-	-	-	-
Concatenation_layer_num	Full connective layer neuron	512	512	-	-	-
Filter_sizes	Convolution kernel size	-	2	-	-	-
Filter_num	Number of convolution kernels	-	256	-	-	-
Cov1D_layer	Convolution layers	-	1	-	-	-
Batch_size	Batch	32	32	-	-	-
Epoches	Epoch	15	15	-	-	-
Dropout	Retention ratio	0.5	0.5	-	-	-
N_estimators	Number of classifiers	-	-	100	-	-
C	Penalty coefficient	-	-	-	-	0.3
Kernel	Kernel function	-	-	-	-	Poly
Gamma	Kernel coefficient	-	-	-	-	0.2

### 2.2. Analysis of the results of the departmental guide recommendations

To verify the classification performance of the model, we selected several indicators to display the results. The specific indicators are shown as follows: When we evaluate the effect of the classification algorithm, we can use precision, recall, f1-score, and accuracy to evaluate the effect of the classification algorithm. Generally, the sample can be classified into positive class (P) and negative class (N). Besides, according to the classifier and actual situation, the sample can be classified into true-positive type (TP), true-negative type (TN), false-positive (FP), and false-negative (FN). This moment, *P* = *TP*+*PN* and *N* = *TN*+*FP*.

(1) Confusion matrix

The confusion matrix is a standard form of accuracy evaluation, which is used to observe the performance of the model in various categories, and calculate the accuracy of the model corresponding to each category. It can reflect the accuracy of model classification effectively and have a good visualization effect in evaluating the classification effect of the model.

(2) Precision

Precision refers to the proportion of samples that are truly positive in the samples that are predicted to be positive, which can indicate the precision of model prediction.


(7) $$\begin{array}{l} Precision=\frac{TP}{TP+FP} \end{array}$$

(3) Recall

Recall indicates the probability that the positive class in the sample is predicted correctly and measures the recall rate of the model.


(8) $$\begin{array}{l} Recall=\frac{TP}{TP+FN} \end{array}$$

(4) F1-score

As the harmonic average of precision and recall, the F1-score can comprehensively reflect the performance of the model by integrating the indicator advantages of precision and recall.


(9) $$\begin{array}{l} F1-score=\frac{2\times Precision\times Recall}{Precision+Recall} \end{array}$$

(5) Accuracy

Accuracy is used to measure the proportion of positive and negative prediction results.


(10) $$\begin{array}{l} Accuracy=\frac{TP+TN}{P+N} \end{array}$$

**Figure 7:**
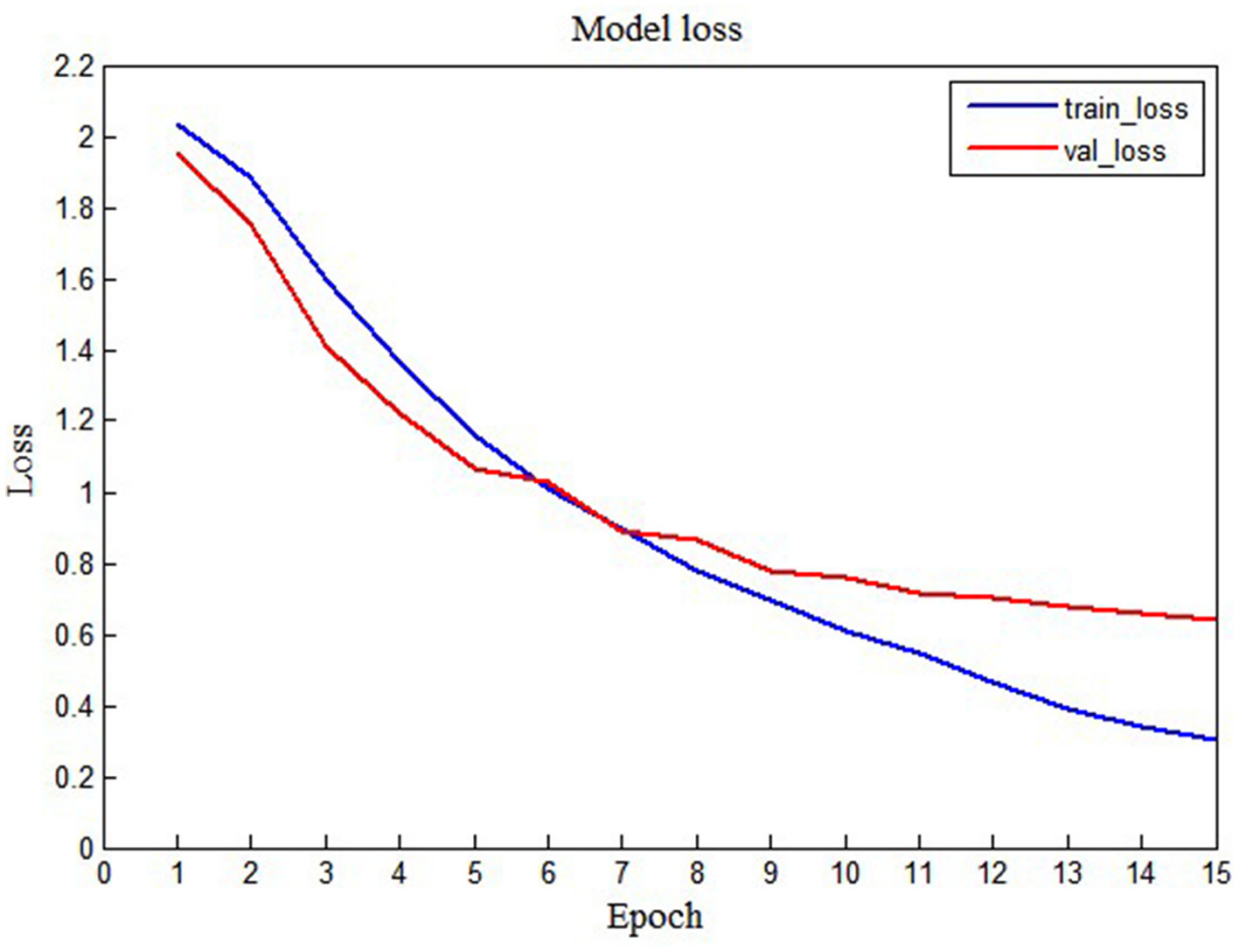
The training loss and validation loss of the LSTM model.

**Figure 8:**
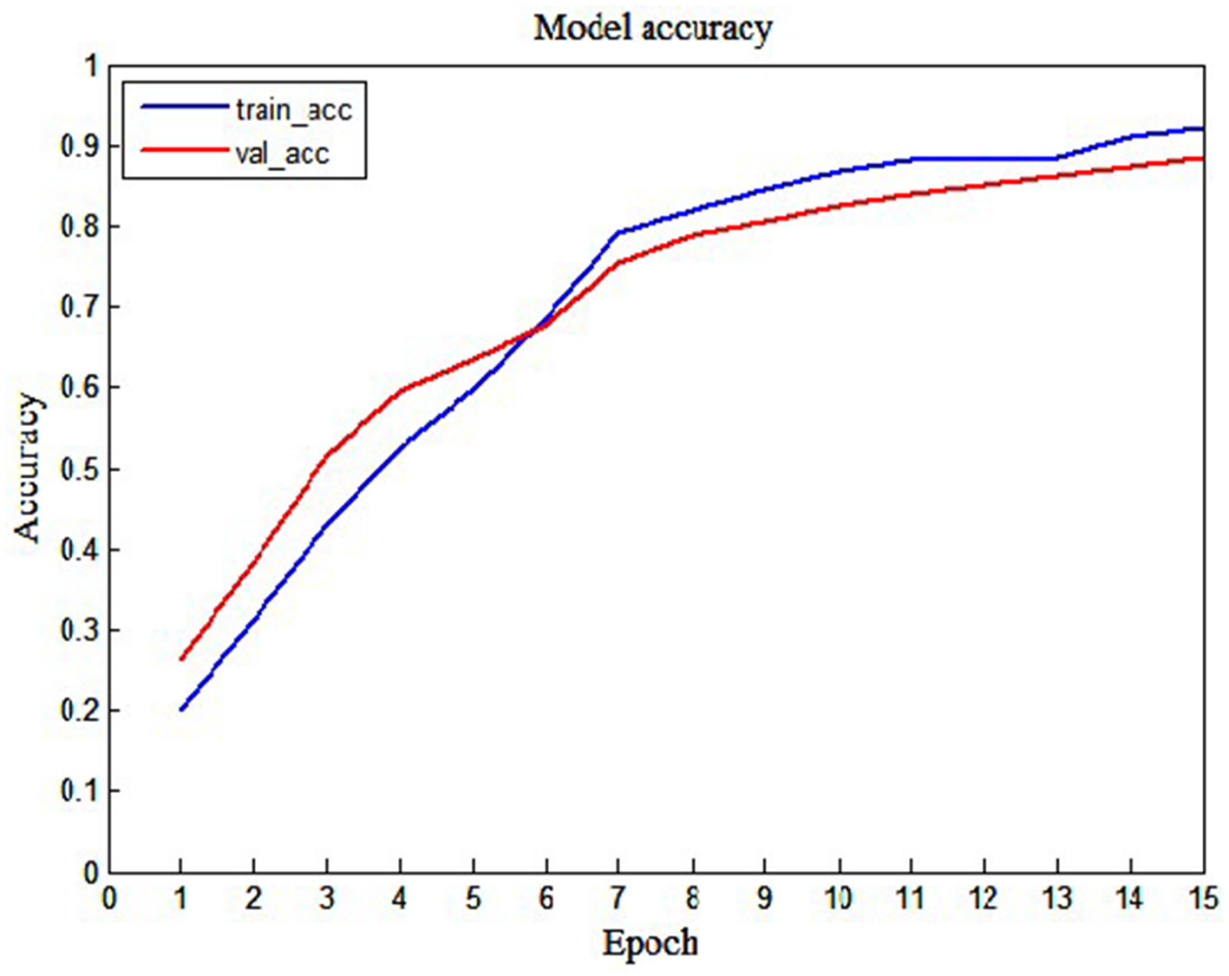
The training accuracy and validation accuracy of the LSTM model.

To display the performance of the LSTM model intuitively, we also show the loss graph and accuracy graph in Figures 7, 8. As shown in Figure 7, we can find that training loss and validation loss decrease gradually. Also, the validation loss decreases more gently in the later stages. The above results show that the LSTM model is learning, and there are no underfitting and overfitting situations. Similarly, as shown in Figure 8, when the epoch increases from 1 to 15, training accuracy and validation accuracy increases from 20% to more than 90%. Thus, it can be seen that the LSTM model has a good learning status.

**Figure 9:**
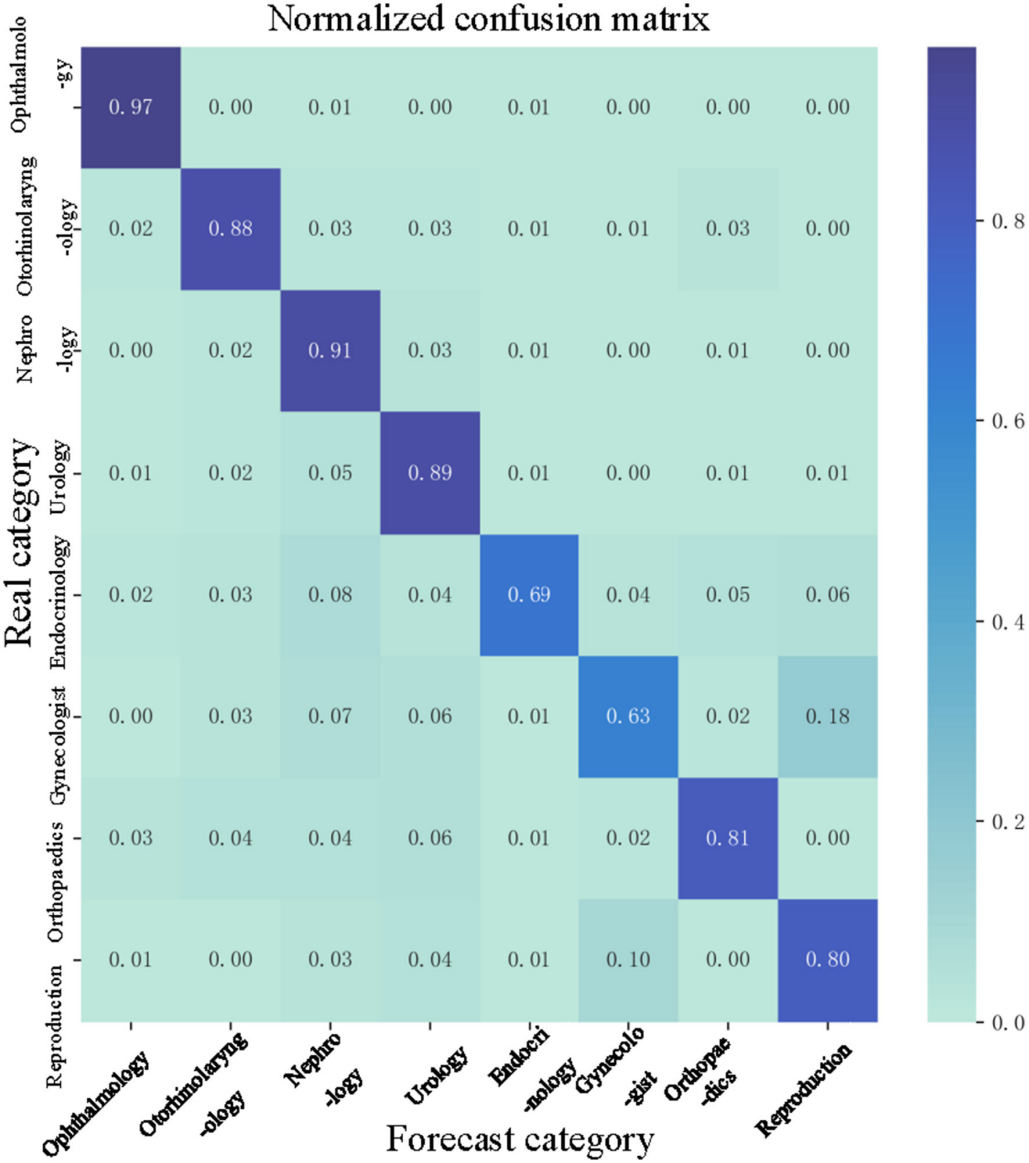
Normalized confusion matrix.

The normalized confusion matrix of the LSTM model is shown in Figure 9. As shown in Figure 9, we can see that the LSTM model can classify the department well. The precision of Ophthalmology is 0.97 and the precision of the Nephrology is 0.91. The precision of a Gynecologist is 0.63, which is the lowest precision. The reason maybe there is a procedure named abortion which could be classified into the Gynecologist, sometimes it could be classified into Reproduction. Therefore, the precision of a Gynecologist is low.

**Figure 10:**
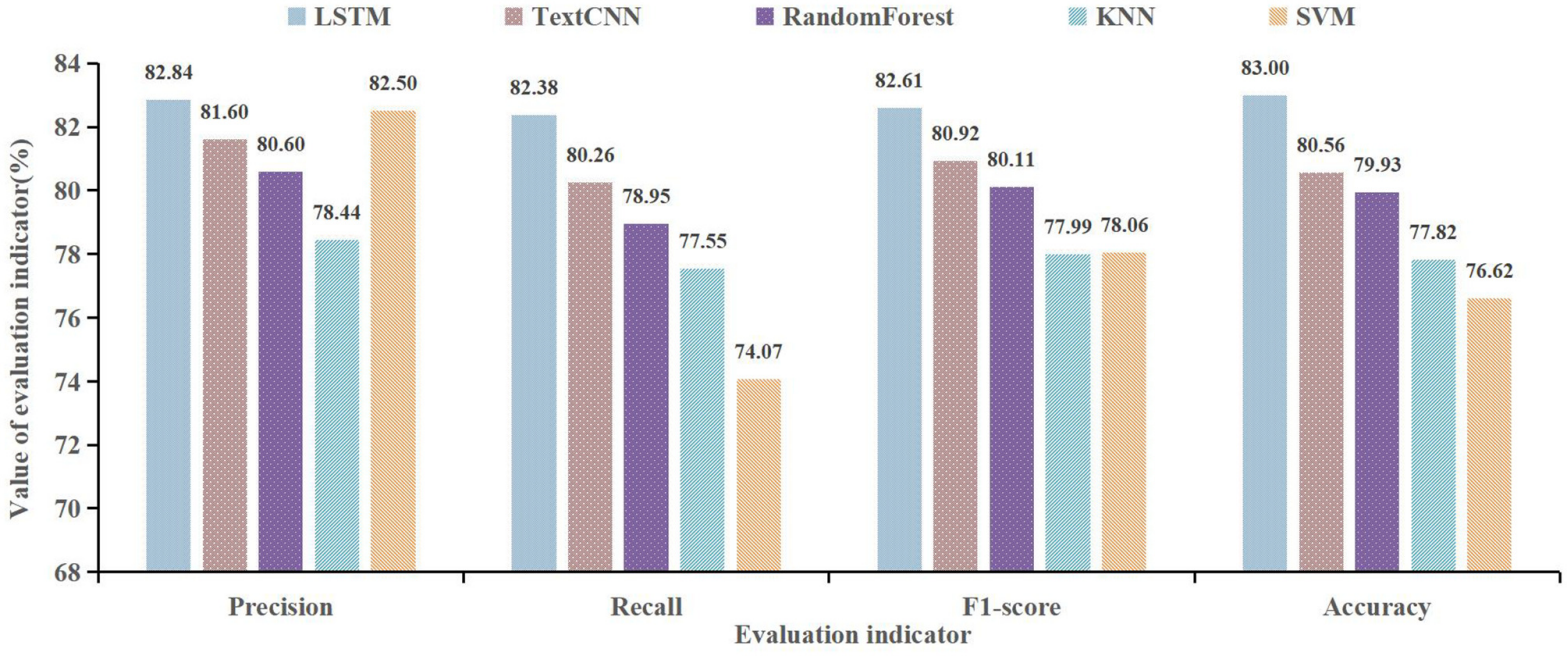
Classification effect of multiple models.

To measure the performance of the LSTM model, we compare the performance of TextCNN, random forest, KNN, and SVM. The specific result is shown in Figure 10.

As shown in Figure 10, we can see that the LSTM model shows the best performance on precision, recall, F1-score, and accuracy. The precision of the LSTM is 82.84%, compared with the TextCNN, random forest, KNN, and SVM, it improved by 1.24%, 2.24%, 4.40%, and 0.34%. The F1-score of the LSTM is 82.61%, compared with TextCNN, random forest, KNN, and SVM, it improved by 1.69%, 2.50%, 4.62%, and 4.55%, respectively. Therefore, the LSTM model has a good performance in department recommendations. In terms of LSTM and TextCNN, we consider that their performance will be dependent on the length and context semantic relationship of data. In this research, the patient's main complaint text is long, so the LSTM can deal with the context semantic relationship well, thus, the LSTM model has a better performance than TextCNN. This result reflects the fact that the structure of the algorithm and the way it is optimized can influence the accuracy of the model effectively. Besides, it can be also found that word vector expression, proportions of training data, and model parameters have an influence on the accuracy of the model.

## 3. Online medical intelligent doctor recommendation model

Based on the department's recommendation, to recommend the appropriate physicians to patients, this study designs an intelligent physician recommendation model further. In our research, we select a department named otorhinolaryngology to carry out the recommendation activity. The data also comes from the Good Doctor, and we use python to crawl diagnosis data and communication data in 63 physicians. One physician can treat multiple patients, and finally, we obtain 1,430 patients' main complaint data and 19,336 communication data. The consulted text from physicians is shown in Table 3.

**Table 3:** The related parameter settings.

**Physician number**	**Physician name**	**Number of texts consulted**	**Consulted text**
1	Weiliang Bai	77	Professor Bai: Hello, my child suddenly said that his left ear hurts and he has a little fever last Wednesday night.
			There is always a foreign body feeling in the throat. The throat always spits out liquid like nasal mucus, and it can't finish spitting.
			What are the four pictures, whether surgery is necessary, and whether conservative treatment is possible?
			...
			After catching a cold, I get angry and have a yellow nose. I don't dare to blow my nose hard. I feel pain when I press the nose. Iodophor disinfection hasn't been good for several days. Can I use any ointment?
2	Yang Zha	25	Do I need an operation? Because I snore seriously. When I open my mouth and breathe my throat is always dry and painful.
			The cold was not treated in time, resulting in nasal congestion. After the cold was cured, nasal congestion still occurred. The doctor required me to do a CT in a local hospital for examination. The doctor diagnosed partial deviation of the nasal septum, hypertrophy of inferior turbinates on both sides, and a little inflammation in the maxillary sinus on both sides.
			I bumped my nose when I was young. I shed a little blood at that time. When I grow up, I often shed nose blood. I can stop bleeding by myself! At present, we are checking for allergic rhinitis and nasal septum deviation!
			...
			For more than a month, I have had a yellow thick nose and a stuffy nose. I have taken clarithromycin and rhinitis tablets repeatedly.
...	...	...	...
63	Kejun Zuo	3	Hello, doctor! I mainly want to consult this time. I take medicine for chronic pharyngitis and rhinitis. But I used the wrong medicine.
			Professor Zuo: Well, CT and nasal endoscopy have been done since the last clinic, and the results have been uploaded. There is also a nasal allergen test report previously examined, with an index of 200+; Please consult and give suggestions. Thank you.
			I have relapsed many times, and I have drawn thick liquid several times. Now I want to have a radical operation. I need an operation urgently. When can I arrange the operation?

The consulted text from patients is shown in Table 4.

**Table 4:** Patients' main complaint text.

**Patient number**	**Consulted text**
1	Professor Bai: Hello, my child suddenly said that his left ear hurts and he has a little fever last Wednesday night.
2	There is always a foreign body feeling in the throat. The throat always spits out liquid like nasal mucus, and it can't finish spitting.
3	What are the four pictures, whether surgery is necessary, and whether conservative treatment is possible?
4	Doctor, I bumped my nose when I was playing football yesterday. I feel uncomfortable. I took CT today.
5	In the past, I snored occasionally. After two times of acute laryngitis in the past 2 months, my snoring and suffocation became worse.
6	When I sleep, I have sudden respiratory arrest, and I wake up with suffocation. My breathing sound is very heavy, and sometimes I snore.
7	The child sleeps half a mouth, does lymphatic swelling need an operation?
8	The child always gasps loudly during sleep and often wakes up in the middle of the night.
9	I have perennial rhinitis. This time, my throat itches, and rhinitis have lasted for 2 months.
10	My mental state is poor, my head is heavy and my heart is anxious. Please help me, doctor.
...	...
1,430	I have recurred many times, and I have drawn thick liquid several times. Now I want to have a radical operation. I need an operation urgently. When can I arrange the operation?

The patient-physician communication text is shown in Table 5.

**Table 5:** Patient-physician communication text.

**Physician** **number**	**Physician** **name**	**Patient** **number**	**Disease** **description**	**Patient** **text**	**Physician** **text**	**Physician** **voice**
1	Weiliang Bai	1	Professor Bai: Hello, my child suddenly said that his left ear hurts and he has a little fever last Wednesday night.		You can use ofloxacin ear drops and oral antibiotics for symptomatic treatment.	
				Antibiotic cephalosporin has been taken for seven days. How many more days can it be taken? How many days does ofloxacin drop? Twice a day? How many drops at a time?		
						10s
				OK, thank you, Professor Bai. Can I also take ear drops for three or five days?		
				...	...	...
		2	I always have a foreign body feeling in my throat. My throat always spits out liquid like snot, and I can't finish it.		Symptoms may be related to nasal septum deviation.	
					If necessary, you will do the nasal septum.	
				Director Bai, is the operation risky?		
						12s
				...	...	...

**Figure 11:**
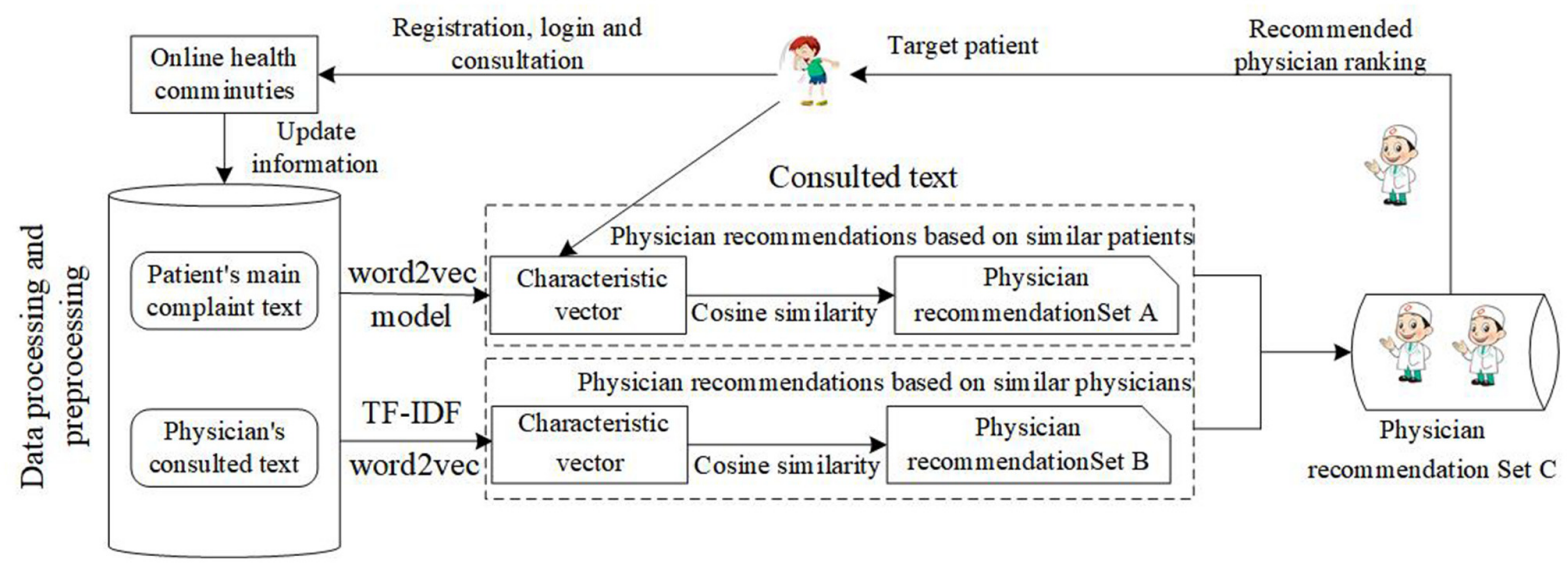
Intelligent physician recommendation process.

Based on the above data, this study designs an intelligent physician recommendation model based on patient-physician interaction data. The specific model is shown in Figure 11.

As shown in Figure 11, when the patients consult in an online health community, the patient's main complaint text and the physician's consulted text are important data sources. Based on training word vectors, we need to use the word2vec model to vectorize the patients' main complaint text in Table 4, and we also need to use TF_IDF to extract keywords from the physician's consulted text. After that, we need to use the word2vec model to vectorize the keywords from the physician's consulted text. Finally, cosine similarity is employed in the recommendation. The cosine similarity is shown as follows:


(11) $$\begin{array}{l} W_{uv}=\frac{\left|N\left(u\right)\cap N\left(v\right)\right|}{\sqrt{\left|N\left(u\right)|*|N\left(v\right)\right|}} \end{array}$$

Where the *u* and *v* indicate patient *u* and patient *v*. *N*(*u*) and *N*(*v*) indicate the word vector set of patient *u* and patient *v*. Besides, in the process of the word vector expression, the patient's complained text is composed of several words. In a sentence, each word is defined as *w*, if there are *k* words, the sentence can be expressed as *d*_*i*_ = *w*_1_, *w*_2_, ..., *w*_*k*_. Furthermore, each word can be mapped to n-dimensional word vectors and can be expressed as *v*_*i*_ = *v*_*i*1_, *v*_*i*2_, ..., *v*_*in*_, the patient's complained text is expressed as follows:


(12) $$\begin{array}{l} d_{i}=\frac{1}{k}\underset{i}{\sum }v_{i} \end{array}$$

### 3.1. Recommendation model based on similar patients

The vector representation of the patient's main complaint text is shown in Table 6.

**Table 6:** The vector representation of the patient's main complaint text.

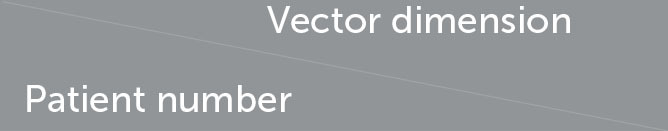	**1**	**2**	**3**	**4**	**5**	**..**.	**256**
1	0.028	–0.049	–0.016	0.113	–0.112	...	–0.042
2	0.005	–0.121	0.033	0.071	–0.066	...	–0.020
3	0.054	–0.037	–0.016	0.240	–0.073	...	–0.018
4	0.099	–0.052	–0.037	0.162	–0.123	...	–0.055
5	0.004	–0.039	–0.015	0.086	–0.042	...	–0.005
6	–0.073	–0.033	0.018	0.020	–0.069	...	–0.001
7	0.029	–0.010	–0.062	0.130	–0.073	...	–0.017
8	–0.025	–0.034	0.059	0.066	–0.031	...	0.045
9	0.026	–0.087	–0.040	0.087	–0.069	...	0.027
10	–0.001	–0.069	–0.036	0.118	–0.036	...	0.006
...	...	...	...	...	...	...	...
1,430	0.022	–0.057	–0.039	0.177	–0.057	...	–0.016

The cosine similarity of the patient main complaint text is shown in Table 7.

**Table 7:** The cosine similarity of the patient's main complaint text.

**Patient number**	**1**	**2**	**3**	**4**	**5**	**6**	**7**	**8**	**9**	**10**	**..**.	**1,430**
1	1.000	0.728	0.837	0.879	0.892	0.653	0.791	0.722	0.870	0.809	...	0.801
2	0.728	1.000	0.579	0.690	0.775	0.694	0.615	0.677	0.818	0.735	...	0.596
3	0.837	0.579	1.000	0.809	0.838	0.529	0.817	0.597	0.715	0.791	...	0.864
4	0.879	0.690	0.809	1.000	0.847	0.643	0.776	0.707	0.791	0.807	...	0.749
5	0.892	0.775	0.838	0.847	1.000	0.808	0.861	0.814	0.892	0.872	...	0.807
6	0.653	0.694	0.529	0.643	0.808	1.000	0.687	0.879	0.748	0.675	...	0.569
7	0.791	0.615	0.817	0.776	0.861	0.687	1.000	0.707	0.745	0.787	...	0.820
8	0.722	0.677	0.597	0.707	0.814	0.879	0.707	1.000	0.770	0.684	...	0.634
9	0.870	0.818	0.715	0.791	0.892	0.748	0.745	0.770	1.000	0.798	...	0.698
10	0.809	0.735	0.791	0.807	0.872	0.675	0.787	0.684	0.798	1.000	...	0.772
...	...	...	...	...	...	...	...	...	...	...	...	...
1,430	0.801	0.596	0.864	0.749	0.807	0.569	0.820	0.634	0.698	0.772	...	1.000

Based on Table 7, we select a patient, for example, a patient numbered 120. We recommend the physician to patient 120. To ensure there is a high similarity of patients, we set the threshold of the cosine similarity to 0.800. If the cosine similarity is larger than 0.800, the physician corresponding to the patient will be recommended in set A. Based on the threshold of the cosine similarity to 0.800, there are 48 physicians will be recommended in set A. The similar patients and physicians of target patient 120 are shown in Table 8.

**Table 8:** Similar patients and physicians of target patient 120.

**Patient number**	**Cosine similarity**	**Physician name**
28	0.943	Weiliang Bai
60	0.935	
43	0.934	
...	...	
9	0.811	
96	0.946	Yang Zha
101	0.922	
100	0.908	
...	...	
78	0.813	
107	0.846	Aiping Chen
106	0.801	
110	0.950	Fenghong Chen
109	0.895	
111	0.893	
120	1.000	Fuquan Chen
115	0.927	
113	0.916	
...	...	
119	0.823	
130	0.903	Jianjun Chen
133	0.901	
132	0.897	
...	...	
135	0.816	
...	...	...
1,430	0.842	Kejun Zuo

Based on Table 8, the top 10 physicians are selected as the physician recommendation set based on similar patients. The 120 target patient recommendation results based on similar patients are shown in Table 9.

**Table 9:** One hundred and twenty target patient recommendation results based on similar patients.

**Patient number**	**Cosine similarity**	**Physician name**
36	0.889	Kaiming Su
24	0.885	Liangfa Liu
5	0.869	Fuquan Chen
4	0.866	Fenghong Chen
63	0.866	Kejun Zuo
19	0.865	Weidong Jin
42	0.865	Min Wang
29	0.859	Cuida Meng
26	0.858	Yongshou Chen
60	0.857	Jin Zhu

### 3.2. Recommendation model based on similar physicians

In this section, we will introduce a recommendation model based on similar physicians. When a patient consults a physician, if the physician is similar to the physician consulted before, we can recommend this physician to the patient. Since the physician's consulted text is a set of multiple patients' main complaint texts, we need to use the TF-IDF to extract the key information, such as the keywords. Term frequency-inverse document frequency(TF-IDF) can represent the eigenvalue weight in the spatial vector model and has a wide range of applications in extracting multi-text features. In this algorithm, the TF-IDF of the keyword *i* in the document *j* is shown in formula (13):


(13) $$\begin{array}{l} tf-idf\left(i,j\right)=tf\left(i,j\right)\times idf\left(i\right) \end{array}$$

Where *tf*(*i, j*) is the word frequency, it indicates the frequency of keyword *i* in document *j*, and *idf*(*i*) is the inverse document frequency. The value of *tf*(*i, j*) is larger, which indicates that the feature value is more important in the document.

In this research, we use the TF-IDF to extract the key information in multiple texts from physicians' consulted text and use the word2vec algorithm to vectorize the key information from the physician's consulted text. Then, these vectors are arithmetically averaged to obtain the final vector expression. To obtain more information from multiple texts, the number of high-frequency words is set to 15. The extracted keywords are vectorized and further averaged to obtain the vector of the physician's consulted text. The vector of the physician's consulted text is shown in Table 10.

**Table 10:** The vector of the physician's consulted text.

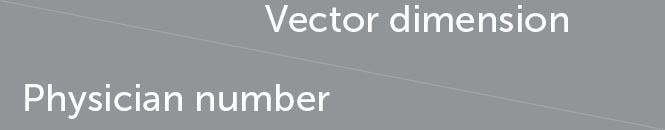	**1**	**2**	**3**	**4**	**5**	**..**.	**256**
1	0.028	–0.049	–0.016	0.113	0.028	...	–0.042
2	0.005	–0.121	0.033	0.071	0.005	...	–0.020
3	0.054	–0.037	–0.016	0.240	0.054	...	–0.018
4	0.099	–0.052	–0.037	0.162	0.099	...	–0.055
5	0.004	–0.039	–0.015	0.086	0.004	...	–0.005
6	–0.073	–0.033	0.018	0.020	–0.073	...	–0.001
7	0.029	–0.010	–0.062	0.130	0.029	...	–0.017
8	–0.025	–0.034	0.059	0.066	–0.025	...	0.045
9	0.026	–0.087	–0.040	0.087	0.026	...	0.027
10	–0.001	–0.069	–0.036	0.118	–0.001	...	0.006
...	...	...	...	...	...	...	...
63	0.040	–0.046	–0.016	0.069	–0.074	...	0.016

Based on the vector of the physician's consulted text, this study calculates the cosine similarity of physicians, the results are shown in Table 11.

**Table 11:** The cosine similarity of physicians.

**Physician number**	**1**	**2**	**3**	**4**	**5**	**6**	**7**	**8**	**9**	**10**	**..**.	**63**
1	1.000	0.728	0.837	0.879	0.892	0.653	0.791	0.722	0.870	0.809	...	0.801
2	0.728	1.000	0.579	0.690	0.775	0.694	0.615	0.677	0.818	0.735	...	0.714
3	0.837	0.579	1.000	0.809	0.838	0.529	0.817	0.597	0.715	0.791	...	0.669
4	0.879	0.690	0.809	1.000	0.847	0.643	0.776	0.707	0.791	0.807	...	0.767
5	0.892	0.775	0.838	0.847	1.000	0.808	0.861	0.814	0.892	0.872	...	0.899
6	0.653	0.694	0.529	0.643	0.808	1.000	0.687	0.879	0.748	0.675	...	0.849
7	0.791	0.615	0.817	0.776	0.861	0.687	1.000	0.707	0.745	0.787	...	0.763
8	0.722	0.677	0.597	0.707	0.814	0.879	0.707	1.000	0.770	0.684	...	0.825
9	0.870	0.818	0.715	0.791	0.892	0.748	0.745	0.770	1.000	0.798	...	0.872
10	0.809	0.735	0.791	0.807	0.872	0.675	0.787	0.684	0.798	1.000	...	0.747
...	...	...	...	...	...	...	...	...	...	...	...	...
63	0.801	0.714	0.669	0.767	0.899	0.849	0.763	0.825	0.872	0.747	...	1.000

The physician consulted by patient 120 is physician 5, whose name is Fuquan Chen. The 10 physicians are most similar to Fuquan Chen, and the recommended result is shown in Table 12.

**Table 12:** One hundred and twenty target patient recommendation results based on similar physicians.

**Physician number**	**Cosine similarity**	**Physician name**
17	0.956	Jiebo Guo
21	0.954	Jiping Li
41	0.945	Jianting Wang
11	0.938	Baocheng Dong
43	0.937	Qi Wang
60	0.937	Jin Zhu
29	0.935	Cuida Meng
57	0.933	Shaoxing Zhang
13	0.929	Yunping Fan
42	0.929	Min Wang

### 3.3. Recommendation model based on similar physicians

In Sections 3.1, 3.2, the recommended physicians based on similar patients form a recommendation set A, and the recommended physicians based on similar physicians form a recommendation set B. Combining sets A and B, we use the similarity in the recommendation set B to sort the recommendation set A and form a new recommendation set C. The recommendation set C is the final recommendation result. The recommended results based on similar patients and similar physicians are shown in Table 13.

**Table 13:** Recommended results based the similar patients and similar physicians.

**Physician number**	**Cosine similarity**	**Physician name**
36	0.889	Kaiming Su
24	0.885	Liangfa Liu
5	0.869	Fuquan Chen
4	0.866	Fenghong Chen
42	0.865	Wen Wang
19	0.865	Weidong Jin
29	0.859	Cuida Meng
60	0.857	Jin Zhu
33	0.854	Wenyu Yu
7	0.854	Jianjun Chen

To test the accuracy of the recommended result, we compare the consult information among recommended results. The disease of patient 120 is ‘In September this year, I gave the children nasal endoscopy and found that 3 / 4 of them were **blocked**. The children **breathed with their mouths open** at night and **snored** occasionally!'. The consultation information of the final recommended result is shown in Table 14.

**Table 14:** Recommended results and diagnosis information of target patient 120.

**Physician name**	**Physician number**	**Physician consulted text**
Kaiming Su	36	The local doctor said that the **congestion** was about 70–80%, and the **breathing was blocked** for about half a year. At this time last year, the nose was **blocked** for several months, and he always thought it was a cold.
		Last year, nasal endoscopy showed that **three-quarters of them were blocked**. This year, only CT was performed. According to the CT doctor, adenoids need surgery.
		I can obviously hear heavy breathing, and I can hear that my nose is **blocked** and I can't breathe.
		Also, when I sleep at night, I **should open my mouth and exhale**. There is no family history.
Liangfa Liu	24	**Dyspnea** occurred last month.
Fuquan Chen	5	The nose is itchy and runny, the mouth is dry, and the throat is itchy. My nose has a bad smell and I had a headache. Go to the local hospital for examination, the doctor says that is **chronic rhinitis**.
		Whenever the flu season occurs in autumn and winter, the nose will be **blocked** for a long time, and the nose will be runny, a little like a cold.
Fenghong Chen	4	When I sleep and breathe, I often feel **nasal mucus**.
Wen Wang	42	The nose on both sides is often **stuffy**.
		I have had allergic rhinitis for many years and often have **nasal congestion**.
Weidong Jin	19	In the follow-up visit on November 29, the result showed that the **congestion** was the same, still 70%.
Cuida Meng	29	At that time, the nasal cavity was **not very smooth**, and then maybe the nasal washing method was wrong.
Jin Zhu	60	The child is five and a half years old, and his nose is basically **stuffy** all year round, especially in winter.
Wenyu Yu	33	The patient had repeated **nasal congestion** and a purulent runny nose for more than 2 years.
Jianjun Chen	7	I started nasal **congestion**, cough, and runny nose.

In Table 14, we can see that the diagnosis information of 10 physicians is similar to the symptoms of patient 120. For example, the diagnosis of physician 36 Kaiming Su contains “**breathing was blocked**” and “**three-quarters of them were blocked**”; the diagnosis of physician 42 Wen Wang contains “**stuffy**,” and “**nasal congestion**.” This diagnosis information is similar to the symptom of patient 120; therefore, the recommended results are relatively accurate.

### 3.4. Analysis and evaluation of recommendation results

In this research, there are 1,430 patients and 63 physicians in the research data. The first recommendation model is the recommended model based on similar patients. In this model, we set the similarity threshold to 0.800, if the similarity between other patients and the target patient is greater than or equal to 0.800, the patients will be taken into the similar patients, then the similarity of these patients treated by one physician was averaged. The physicians ranked in the top 10 in the similarity will be taken as the final recommendation results. The second model is the recommendation model based on similar physicians. By calculating the similarity between the remaining 62 physicians and the physician consulted by the target patient, the top 10 physicians were selected as the final recommendation results. The third model is the recommendation model based on similar patients and physicians. In this model, we combined the results of similar patients and physicians to sort further, if the physicians in the model of similar patients are not in the list of the model of similar physicians, the physicians will be deleted and the next physician will fill that position, finally, we can get the final top 10 results.

To build a good evaluation indicator, the evaluation indicator should be composed of three indicators: recommendation precision, text service quality, and voice service quality. If the number of consultation texts that is similar to the patient's consultation text in the recommended physician accounts for 80% or more, the recommendation is deemed to be successful. The recommendation precision is the proportion of the physician who is recommended successfully to the total recommended numbers. In the real life, in addition to the recommendation precision, service quality is also an important indicator. In online health communities, if the physicians speak more text or voice information, the patients will consider that the physician's service is better than the physician who says fewer words or voice, therefore, the text and voice service quality are two important evaluating indicators. The specific calculation method of text and voice service quality is shown as follows: First, we need to calculate the average text and voice length of 63 physicians in our data set. Second, comparing the text length and voice length of recommended physicians with the average of total physicians. If the text and voice length of recommended physicians is larger than the average length, the text and voice service quality is well and the recommended physician in the aspect of text and voice service quality is successful. For example, if 10 physicians are recommended, there are 5 physicians' text length that is larger than the average length of 63 physicians, and the text service quality is 0.5. Similarly, the voice service quality is similar to the text service quality. The final recommendation performance (*RP*) is a linear weight among recommendation precision, text service quality, and voice service quality. To sign the indicator conveniently, we set the recommendation precision as *A*, set text service quality as *B*, and set voice service quality as *C*. The weight of these factors is τ_1_, τ_2_, and τ_3_. Therefore, the *RP* is a linear weighted function and it can be expressed as follows:


(14) $$\begin{array}{l} RP=\tau_{1}A+\tau_{2}B+\tau_{3}C \end{array}$$

In the recommendation model, the first factor considered by the patients is the recommendation precision. If the system can recommend a professional and suitable physician to patients, the patients will be satisfied. Then, the text and voice service quality will be considered. Therefore, we can set the weight of the recommendation precision to be larger and set the weight of the text and voice service quality to be smaller. Without losing generality, we set the original weight to τ_1_ = 0.8, τ_2_ = 0.1, and τ_3_ = 0.1. Besides, the similarity threshold and recommended success rate are two important factors that can influence the final recommendation precision and the text and voice service quality. To write conveniently, we set the similarity threshold as β and set the recommended success rate as γ. To test the performance of the three recommendation models, namely recommendation models based on similar patients, recommendation models based on similar physicians, and recommendation models based on similar patients and physicians. Furthermore, we set different parameters under different conditions to examine the performance of the three models. The recommendation performance of the three recommendation models is shown in Table 15.

**Table 15:** Recommendation performance of three recommendation models under different parameters.

**Parameter setting**		**Similar patients**	**Similar physicians**	**Similar patients and physicians**
		*RP*	*RP*	*RP*
β = 0.5	γ = 0.5	0.8496	0.8420	0.8495
	γ = 0.6	0.8477	0.8199	0.8476
	γ = 0.7	0.8456	0.7974	0.8455
	γ = 0.8	0.8444	0.7932	0.8443
	γ = 0.9	0.8403	0.7748	0.8401
β = 0.6	γ = 0.5	**0.8390**	0.8006	**0.8396**
	γ = 0.6	**0.8352**	0.7746	**0.8357**
	γ = 0.7	**0.8287**	0.7283	**0.8291**
	γ = 0.8	**0.8239**	0.7104	**0.8243**
	γ = 0.9	0.8054	0.7196	0.8043
β = 0.7	γ = 0.5	**0.8033**	0.8420	**0.8043**
	γ = 0.6	0.7907	0.6769	0.7905
	γ = 0.7	0.7702	0.5986	0.7681
	γ = 0.8	0.7405	0.5408	0.7347
	γ = 0.9	0.6768	0.3856	0.6559
β = 0.8	γ = 0.5	**0.6562**	0.4951	**0.6574**
	γ = 0.6	0.6145	0.4064	0.6105
	γ = 0.7	0.5521	0.2929	0.5425
	γ = 0.8	0.4553	0.2287	0.4297
	γ = 0.9	0.3438	0.1554	0.2839
β = 0.9	γ = 0.5	0.1641	0.1106	0.1641
	γ = 0.6	0.1191	0.0899	0.1191
	γ = 0.7	0.0994	0.0758	0.0994
	γ = 0.8	0.0767	0.0756	0.0767
	γ = 0.9	0.0742	0.0753	0.0742

As shown in Table 15, under different parameters, the recommendation based on similar patients and physicians have certain advantages. For example, when β = 0.6, γ = 0.5, γ = 0.6, γ = 0.7, γ = 0.8; β = 0.7, γ = 0.5; β = 0.8, γ = 0.5, the performance of the recommendation model based on similar patients and physicians has certain advantages, compared to the recommendation model based on similar patients and the recommendation model based on similar physicians.

## 4. Discussion

As a novel field of intelligent technology, intelligent medical guidance and recommendation have huge advantages in the medical field (33, 34). At present, previous research has explored the intelligent department recommendation and physician recommendation model separately (35–37), there is spare research exploring the intelligent department guidance and physician recommendation model systematically. Besides, with the development of online health communities, patient-physician communication data have huge value. Based on this, to the best of our knowledge, our research on intelligent medical guidance and recommendation was the earlier research based on patient-physician communication data. In this research, to solve the problem that the patients know the diseases but do not know the department and the patients know the department but do not know how to select a good physician to consult, this study constructs an intelligent medical guidance and recommendation model. The model contains two parts of contents: one is the intelligent department recommendation and the other is the intelligent physician recommendation.

First, in the model of intelligent department recommendations, based on the long text characteristic of patient-physician communication, due to the advantage of processing the context semantic relationship (38), we use the LSTM algorithm to construct an intelligent department recommendation model. Compared with TextCNN, random forest, KNN, and SVM algorithms, the LSTM model has a certain advantage in department recommendation. In terms of a specific indicator, the precision of the LSTM is 82.84% compared with TextCNN, random forest, KNN, and SVM algorithms, it improved by 1.24%, 2.24%, 4.40%, and 0.34%. The F1-score of the LSTM is 82.61% compared with TextCNN, random forest, KNN, and SVM algorithms, it improved by 1.69%, 2.50%, 4.62%, and 4.55%. Therefore, the LSTM model has a good performance in department recommendations.

Second, we use the text analysis method to construct the recommendation model of similar patients and similar physicians. In this part, the TF-IDF, word2vec, and cosine similarity were applied to our research. Based on the patient-physician communication data, we build physician recommendation models based on similar patients, similar physicians, and similar patients and physicians. Distinct from previous studies, with respect to evaluating indicators, in addition to recommendation precision, we introduce the text and voice service quality to the evaluating indicator. Under certain parameters, we found that the performance of recommendation based on similar patients and physicians is better than the recommendation model based on similar patients and similar physicians separately. The model can help patients to find the appropriate physicians to consult.

Third, the intelligent medical guidance and recommendation model is constructed in our research. Although previous studies have explored the intelligent department recommendation and the intelligent physician recommendation separately, few studies designed a systematic intelligent medical guidance and recommendation model that help patients to find the right department and the right doctor. Our research uses text analysis and machine learning to construct an intelligent department recommendation model and an intelligent physician recommendation model. These models can help patients find the appropriate department and physician, especially if it is of great use to the elderly.

## 5. Limitations and future directions

Although our research explored intelligent medical guidance and recommendation model, there are still some limitations in this research. First, limited by the availability of data, our data comes from a single online health community named Good Doctor, not multiple platforms. The single data sources may make the recommendation efficiency not very high. In the future, we will select more data to train and update the recommendation model.

Second, as a recommendation model, to some degree, our research is confronted with the cold-start problem. In the intelligent department recommendation model, if there are no similar symptoms to the new patient in the database, the recommendation efficiency will be reduced or even unable to recommend. Similarly, in the intelligent physician recommendation model, if the patients have an unusual disease, it is also difficult to make good recommendations to patients. Therefore, in the future, the database needs to be extended by introducing more data to realize an efficient and accurate recommendation.

Third, although we use text analysis and machine learning to construct the recommendation model, the model and method still have some limitations. For example, although we train the word vector model with a medical corpus, our corpus is still small. Future studies can select more medical corpus to express the text precisely. Besides, on the method, we use the LSTM to do a department recommendation. With the development of deep learning, future studies can use more efficient algorithms for department recommendation research, such as the BERT and transformer algorithms.

## 6. Conclusion

As a new technology, intelligent recommendation brings great convenience to people's life. However, in medical fields, the application of intelligent medical recommendation systems is still less, especially the system based on patient-physician communication data. Due to the fact that there is information asymmetry between doctors and patients, patients know their diseases but do not know how to select an appropriate department to consult, and even though they know which department they do not know select which physician. Based on this, we use text analysis, natural language processing, and machine learning to construct an intelligent department recommendation model and an intelligent physician recommendation model. In the intelligent department recommendation model, considering that the patient's main complaint text is long and has context semantic relevance, we use the LSTM model to construct a department recommendation model. Compared to TextCNN, random forest, KNN, and SVM algorithms, the LSTM algorithm has certain advantages in each evaluation indicator. The precision of LSTM is 82.84% and the F1-score is 82.61% in the test set.

In the intelligent physician recommendation model, considering patient and physician similarities, this study also designs a physician recommendation model based on similar patients and similar physicians. Meanwhile, in terms of evaluation indicators, in addition to the recommendation precision, this study also introduces text service quality and voice service quality to the evaluation indicator. Compared with the recommendation model based on similar patients and the recommendation model based on similar physicians separately, the physician recommendation model based on similar patients and similar physicians have better performance. Overall, this research can give academic circles and hospitals some enlightenment and can provide some references for the reasonable matching between physician and patient resources.

## Data availability statement

The raw data supporting the conclusions of this article will be made available by the authors, without undue reservation.

## Ethics statement

The data used in the manuscript is publicly available on the website of Good Doctor Online. The data provided by the platform are all anonymous and any information related to personal privacy has been removed.

## Author contributions

JL: conceptualization, methodology, writing–original draft, and investigation. CL: methodology, supervision, and validation. YH: methodology and investigation. JH: funding support and validation. All authors contributed to the article and approved the submitted version.
